# Heterogeneity of Orodental Features in a Family with Noonan Syndrome

**DOI:** 10.3390/ijms262311414

**Published:** 2025-11-26

**Authors:** Gréta Antal, Laura Csabai, Anna Zsigmond, Ildiko Szanto, Kinga Hadzsiev, Judit Bene

**Affiliations:** 1 Department of Dentistry, Oral and Maxillofacial Surgery, Clinical Center, Medical School, University of Pécs, 7624 Pécs, Hungary; antal.greta@pte.hu (G.A.); csabai.laura@pte.hu (L.C.); szanto.ildiko@pte.hu (I.S.); 2 Department of Medical Genetics, Clinical Center, Medical School, University of Pécs, 7624 Pécs, Hungary; zsigmond.anna@pte.hu (A.Z.); hadzsiev.kinga@pte.hu (K.H.)

**Keywords:** Noonan syndrome, RASopathies, genetic syndrome, *PTPN11* gene, oral manifestations

## Abstract

Noonan syndrome is a relatively common genetic syndrome with clinical and genetic heterogeneity. Besides the characteristic features such as short stature, typical facial features, congenital heart defects, skeletal and ocular anomalies, various orodental manifestations occur with variable frequency. High-arched palate, malocclusions, micrognathism, giant cell lesions, and anomalous lateral incisors are frequently observed features, whereas supernumerary teeth, hypodontia, macrodontia, enamel hypoplasia, severe dental caries, impacted teeth, delayed eruption, taurodontism and odontoma have occasionally been reported. Here, we present a family with three affected members displaying variable dental manifestations carrying the same *PTPN11* c.178G>A pathogenic variant. A 14-year-old and a 12-year-old, both female patients, presented high-arched palates, delayed dental eruption and caries. Moreover, the younger sibling exhibited frequently observed manifestations such as malocclusion and gingivitis, and further rare features like open-bite, micrognathia, and crowded teeth were present. The mother of the patients had periodontitis and enamel problems. Monitoring the oral health of the patients with NS is important, as they are prone to severe dental caries, gingival and other orodental problems. Therefore, initiating early orodental examination is highly recommended for patients with suspicion or diagnosis of NS.

## 1. Introduction

Noonan syndrome (NS) is a relatively common, highly variable congenital disorder affecting multiple organ systems. It shows mostly autosomal dominant inheritance and has an estimated incidence of about 1 in 1000–2500 live births, without race and gender predilection. Clinical and genetic heterogeneity is characteristic of this syndrome, which belongs to RASopathies. Nearly 30–75% of the patients have a positive family history [1,2,3], while in many patients, a de novo pathogenic variant occurs [4].

The clinical hallmarks of NS are typical facial features, short stature (50–60%), congenital heart defects, including pulmonary stenosis (50–62%) and hypertrophic cardiomyopathy (HCM, 10–20%), skeletal anomalies (i.e., chest deformities (70–95%) and scoliosis (10–15%)) [5], cryptorchidism in males (60–77%), ocular abnormalities, cognitive deficits of variable degree and bleeding abnormalities (20%) [5,6]. The craniofacial manifestations may comprise relative macrocephaly, triangular facial appearance, downslanting palpebral fissures, epicanthal folds, hypertelorism, ptosis, low-set posteriorly rotated ears and short webbed neck [7,8]. In addition, patients with NS have an increased risk of developing various solid tumors, such as multiple giant cell lesions, embryonal rhabdomyosarcoma, cutaneous tumors and blood cancers [9].

NS is caused by germline mutations in genes involved in the RAS/mitogen-activated protein kinase (MAPK) signal transduction pathway, which plays an essential role in various cellular processes, including proliferation, differentiation, migration, survival and metabolism [10]. Pathogenic mutations in more than 14 genes have been linked to NS. *PTPN11* gene is the major causative gene; about half of the patients possess mutation within this gene. In addition, mutations in the *SOS1*, *RAF1*, *KRAS*, *NRAS*, *BRAF*, *SHOC2*, *NF1*, *MAP2K1*, *RIT1*, *SOS2*, *A2ML1*, *CBL*, and *LZTR1* genes have also been implicated in the pathogenesis of NS [10,11].

Various orodental manifestations may appear in the clinical picture of Noonan syndrome, but limited data is available on it in the literature. These features are micrognathia, high-arched palate, enamel hypoplasia, dental malocclusion, delayed eruption, impacted teeth, hypodontia, supernumerary teeth, taurodontism and odontoma [12,13,14,15].

In this case report, we describe a family with three affected members with variable dental characteristics carrying the same pathogenic *PTPN11* gene variant. In our study, we aim to highlight the variability of dental features not only within the population of NS patients but also within a family.

## 2. Case Presentation

**Figure 1:**
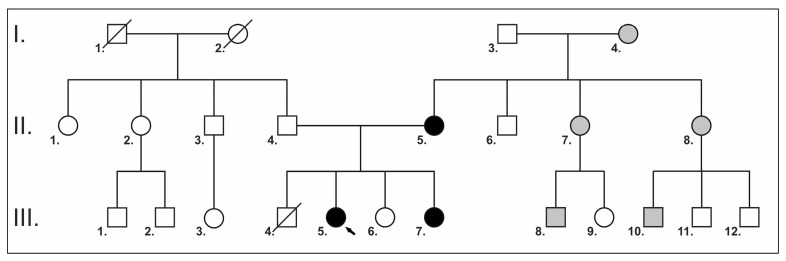
A pedigree of the family. Squares indicate males, and circles indicate females. Symbols in black represent affected subjects; clear symbols represent unaffected subjects; and crossed symbols represent deceased family members. The black arrow indicates the proband. Gray squares and circles indicate potential family members with NS, but clinical and genetic testing has not been performed.

**Figure 2:**
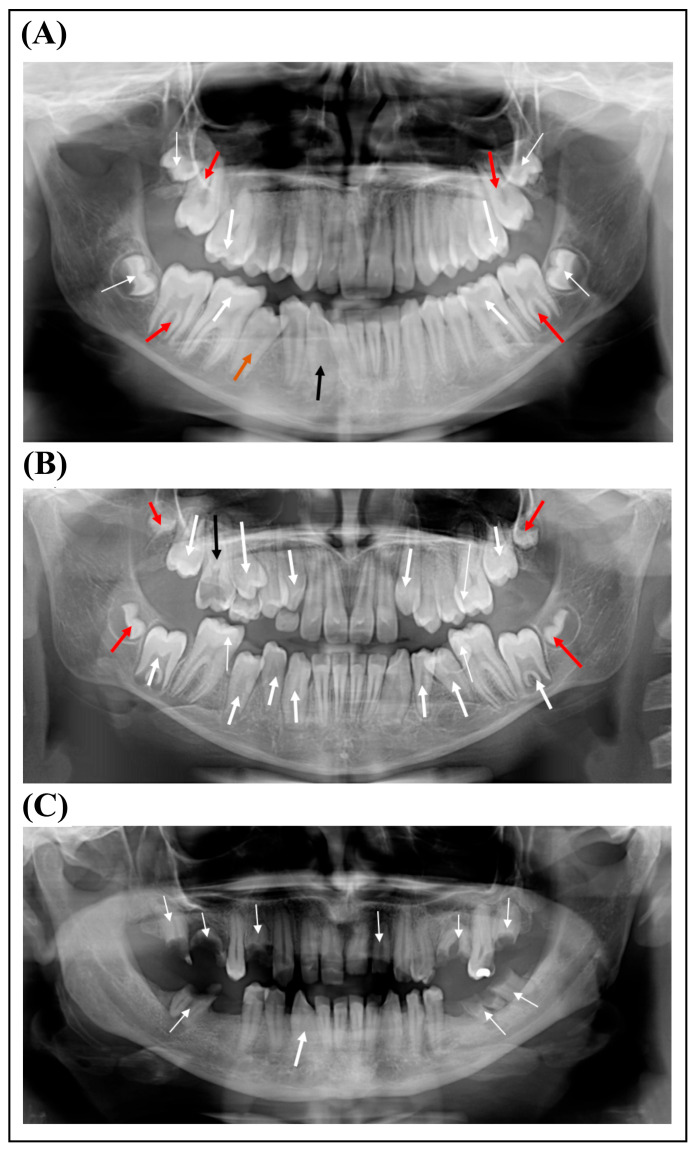
(**A**) Panoramic radiograph of the proband shows occlusal caries (thick white arrows), delayed permanent teeth eruption of the second molars (red arrows) and rotation of tooth 43 (thin black arrow). Thin white arrows indicate the unerupted third molars, and the orange arrow indicates the second premolar being erupted. (**B**) Panoramic radiograph of the proband’s sister shows several teeth (13, 14, 23, 34, 35, 43, 44 and 45) in the process of eruption (thick white arrows). Thin white arrows indicate dental caries, and black arrow indicates root remnant of tooth 16 and red arrows indicate the unerupted third molars. (**C**) Panoramic radiograph of the proband’s mother. Thin white arrows indicate root remnants and the thick white arrow indicates the rotation of tooth 43.

An 11-year-old female patient (proband, P1) was referred to the Genetic Counseling Unit of our Department by the Endocrinology Department of the Paediatric Clinic, University of Pécs due to short stature. She was the second child of non-consanguineous parents (Figure 1) and was born from an uneventful pregnancy at the 37th week of gestation with a birth weight of 2520 g and birth length of 52 cm. Her psychomotor development was normal. She does not have hearing impairment, cardiac involvement, skeletal anomalies and she has no history of bleeding abnormalities. However, she has been under ophthalmological care due to hypermetropia since the age of 3 years. Although her intelligence is normal, she has special educational needs. She has proportionately short stature (below third percentile), and her bone age is delayed by 2.5 years. Extra-orally, at the age of 11, the patient presented with characteristic facial dysmorphic features of prominent forehead, protruding ears and pointed nose with broad nasal tip. The intra-oral examination was performed by board-certified pediatric dentists at the Department of Dentistry and Oral Surgery, University of Pécs. The occurrence of marginal gingival redness, gingival bleeding, enlarged gingiva and gingival sensitivity indicated gingivitis. The other dental, mucosal and skeletal pathologies of the oral cavity were also evaluated mainly by visual assessment. At the age of 14, the intra-oral examination revealed a permanent dentition with good oral hygiene and a high-arched palate. She had delayed eruption of primary teeth; her first tooth erupted at the age of 16 months. Panoramic X-ray (Figure 2A) showed a delayed permanent teeth eruption of the second molars, occlusal caries of the first molars and rotation of tooth 43.

Her family history shows that her mother, one of her sisters, two cousins, two maternal aunts and her maternal grandmother were also short. Her preterm brother died at the age of 11 weeks; he was born with a large cyst on his neck. The family pedigree is shown in Figure 1.

The affected younger sister of our proband (P2) presented to our Genetic Counselling Unit at the age of 8.5 years. She was born on the 37th week of gestation. Her birth weight was 2360 g and her length was 46 cm. Her early psychomotor development was slightly delayed. Her intelligence is normal; however, she has learning difficulties. She has worn glasses since the age of two due to hypermetropia. Her stature is proportionally short (<3 pc), and her bone age is delayed by 2 years. Cardiological examination did not reveal any structural abnormality. She does not have skeletal anomalies, and she has no history of bleeding disorders. Extra-orally, at the age of 8.5, she exhibited a prominent forehead, hypertelorism, low-set protruding ears and broad nasal tip. She was in mixed dentition and her oral hygiene was good; she brushed her teeth twice daily. The intra-oral examination performed by board-certified pediatric dentists at the age of 12 revealed a Class I malocclusion with crowded teeth, high-arched palate, open-bite, micrognathia, hypoplastic jaw and marginal gingivitis. She had delayed eruption of primary teeth, and her first tooth erupted at the age of 16 months. Panoramic X-ray (Figure 2B) showed a delayed permanent teeth eruption of the mandibular right canine, mesial and occlusal caries of teeth 26, 36, 46 and 55 and root remnant of 16.

The mother of the sisters is also short. She has been continuously affected by iron-deficiency anemia since the age of 17 and requires transfusions after surgery. She has prolonged menstrual bleeding and cardiac arrhythmia. The intra-oral examination revealed a permanent dentition with poor oral hygiene and generalized gingival inflammation. Moreover, she had periodontitis and enamel problems. Panoramic X-ray (Figure 2C) showed several missing teeth, a rotation of tooth 43 and root remnant of molars and teeth 14 and 22.

**Figure 3:**
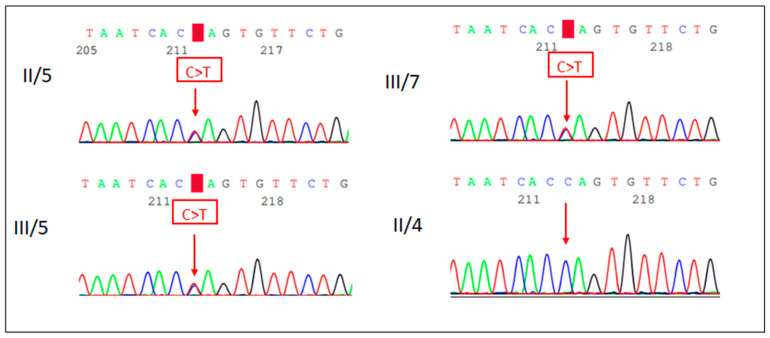
Sanger sequencing electropherogram (reverse strand) of the proband (III/5), her sister (III/7) and her parents (II/5 and II/4). The proband, her sister and her mother carry the *PTPN11* c.178G>A p.(Gly60Ser) missense variant.

A Next-Generation Sequencing (NGS)-based “Comprehensive Growth Disorders/Skeletal Dysplasias and Disorders” gene panel analysis of 510 genes was performed in the proband’s DNA in an international collaboration (Blueprint Genetics laboratory). The test identified a known pathogenic variant, the c.178G>A p.(Gly60Ser) missense variant in the *PTPN11* (NM_002834.4) gene in heterozygous form. Targeted mutation analysis by Sanger sequencing in the samples of the proband’s younger sibling and her mother also detected the mutation in heterozygous form (Figure 3). The phenotype of the patients and their genetic test results confirmed the diagnosis of NS.

## 3. Discussion

Noonan syndrome (OMIM 163950) is a relatively common genetic disorder and it was first described by Noonan and Ehmke in 1963 [16]. Noonan syndrome is characterized by the triad of short stature, craniofacial dysmorphism and congenital heart defects [16]. The facial features are most characteristic in infancy and early to middle childhood; however, in adulthood, they become more subtle. It is proposed that the orofacial features of NS are due to edema of the face and neck, which is a consequence of developmental disturbances of the third and fourth pharyngeal arches [17].

Besides typical facial features, individuals with NS may display various oral manifestations. Although not all the patients have the same oral symptoms, there are frequently observed manifestations such as high-arched palate, malocclusions, micrognathism, giant cell lesions and anomalous lateral incisors. In addition, supernumerary teeth, hypodontia, macrodontia, enamel hypoplasia, severe dental caries, impacted teeth, delayed eruption, taurodontism, odontoma, agenesis and dystrophy have also been occasionally reported [18].

Majority of the intra-oral characteristics of NS are known from individual case reports; only a few studies presented data for a series of patients with NS. In their study, Gürsoy [19] and colleagues investigated the orodental manifestations along with the genetic analyses of 29 patients. Orodental examination was performed in 17 patients. They found that the most common orodental feature was high-arched palate (76.4%), followed by gingivitis (35.2%) and severe caries (35.2%). Moreover, they detected anterior open-bite (11.7%), posterior crossbite (17.6%) and prognathism (17.6%) in their patient cohort. Uncommon findings were hypodontia (11.7%), macroglossia (11.7%) and gingival hyperplasia (5.8%). One patient did not have any orodental manifestations, whereas five patients presented only with high-arched palate. In addition, eleven patients displayed more than one orodental finding. The genetic analysis of the total patient cohort (29 individuals) revealed mutation in the *PTPN11* gene in 89.6% of the patients, and mutation in the *SOS1* gene in three patients. Mallineni and coworkers [20] presented four cases and they found malocclusion, high-arched palate, crowded teeth, impacted teeth and taurodontism as common features. In addition, micrognathia, hypodontia and gingivitis were frequently observed. Moreover, macrodontia, enamel hypoplasia and double tooth were also noted. A retrospective multicenter study was conducted by Lutz and colleagues [13]. Genetic analysis of 14 patients was performed, which revealed causative mutation in 10 patients. Intra-oral manifestations, including malocclusions (maxillary transversal deficiency, crossbite, anterior open-bite and class II malocclusion), dental anomalies (delayed eruption, agenesis, dystrophy and odontoma) and radiologic jaw lesions were identified in 5 out of 10 patients. Malocclusion was the most common finding among these patients; majority of the orodental anomalies were present in only one single patient. Genetic analysis detected mutations in the *PTPN11* gene in 50% of the patients and two patients carried a mutation in *SOS1* gene [13]. An intra-oral clinical evaluation along with radiographic analysis and microbiological profiling were carried out in a series of 11 patients with NS [18]. The authors observed that only very few dental findings were exhibited in their patients compared to previously reported findings. Gingivitis and carious lesions were present in all subjects and could be related to poor oral hygiene. Moreover, high-arched palate (91%) and malocclusions (64%) such as crossbite, open-bite, and deep-bite were frequently observed and two patients presented crowded teeth. Janas-Naze and colleagues [21] investigated the genetic and surgical data of 42 children retrospectively. *PTPN11* mutations were found in 17 patients; 7 individuals carried an *SOS1* gene mutation, and in 12 patients, a mutation in the *LZTR1* gene occurred. Genetic data of eight patients were not available. Patients with *PTPN11* mutations were diagnosed with over-retained deciduous teeth and supernumerary teeth, whereas patients with *SOS1* mutation were diagnosed with mandibular compound odontomas and individuals with *LZTR1* mutations with bilateral or unilateral central giant cell granulomas in the mandible. Relatively few dental features were investigated, and among them, retained deciduous teeth occurred in 24% of the patients, while supernumerary teeth were found in 26% of the cases.

Consistent with the literature, our 14-year-old and 12-year-old female patients exhibited delayed dental eruption, high-arched palate and caries (Table 1). In addition, malocclusion, open-bite, crowded teeth, micrognathia, gingivitis and hypoplastic jaw were also observed in one of our patients. However, hypodontia, supernumerary teeth, impacted teeth, taurodontism and enamel hypoplasia were not found. The mother of the patients showed some oral manifestations such as periodontitis and enamel problems; however, poor oral hygiene alone or superimposed with the *PTPN11* mutation might have led to these oral features. Surprisingly, in our presented family, the affected family members exhibited different orodental anomalies though they carried the same *PTPN11* gene mutation. The *PTPN11* c.178G>A pathogenic variant demonstrates variable expressivity, which significantly influences the phenotype in affected individuals. This could explain the different dental manifestations observed within the same family. Intrafamilial clinical heterogeneity is a known phenomenon in several genetic diseases, among others, and in Noonan syndrome as well [22,23,24]. However, according to our knowledge, this is the first paper presenting intrafamilial variability related to dental features in Noonan syndrome. Intrafamilial clinical heterogeneity makes diagnosis and genetic counseling difficult in these cases.

*PTPN11* gene is located on chromosome 12q24.1, encodes the nonreceptor protein tyrosine phosphatase (PTP) SHP-2 and consists of 16 exons. The SHP2 protein contains two tandem SH2 (Src homology 2) domains at the N terminus (N-SH2 and C-SH2), a catalytic tyrosine phosphatase domain (PTP) and a proline-rich C-terminal tail [25,26]. The N-SH2 domain interacts directly with the PTP domain and acts as a molecular switch between active and inactive conformation of the SHP-2 protein. Several mutations associated with NS affect this interaction, destabilizing the catalytically inactive conformation of the protein. This leads to a gain of function (GOF) of SHP-2 [3,25]. The c.178G>A variant detected in our patients is located in exon 2, results in the Gly60Ser substitution in the N-SH2 domain. This variant is similar to other GOF mutations, and is supposed to cause SHP-2 to be constitutively active by disrupting its auto-inhibited state, leading to enhanced activation of downstream pathways like the Ras-ERK and PI3K-Akt pathways.

The oral manifestations in NS may lead to feeding difficulties and facial growth problems; therefore, a multidisciplinary team care may be required. According to the guidelines for the management of NS, the first dental assessment should be between 1 and 2 years of age, then an annual dental check-up is highly recommended to prevent problems from turning irreversible [9,19,20]. Monitoring the oral health of the patients with NS is important, as they are prone to severe dental caries, gingival and other orodental problems. It is important to prevent caries in early childhood in order to avoid subsequent problems during the eruption of the permanent teeth [20,27]. Therefore, patients with suspicion or diagnosis of NS should be examined orodentally.

Many genetically determined diseases have dental manifestations [28]. Approximately 900 out of 5000 genetic syndromes had dental or maxillofacial anomalies in the clinical pictures according to the London Dysmorphology Database in 2011 [29]. In certain cases, dental and oral anomalies, together with extraoral symptoms, can facilitate the early diagnosis of genetic syndromes [30]. The early diagnosis of NS is essential due to its association with general and oral health [31]. Due to modern genetic testing methods, such as NGS-based targeted sequencing or whole exome sequencing, molecular genetic diagnosis is possible in the majority of these syndromes.

The diagnosis of NS is challenging due to clinical heterogeneity and the prevalence may be underestimated in the population. The early diagnosis of the syndrome is particularly important as it can cause severe manifestations such as cardiac malformations, bleeding abnormalities and an increased risk of the development of various solid tumors. Furthermore, NS can be associated with severe dental complications. Genetic testing is an essential tool in early diagnosis, which helps to introduce early intervention, leading to the improvement in the quality of life. Close dental monitoring is recommended in patients without dental symptoms but carrying a disease-causing genetic variant to prevent possible severe dental manifestations.

## 4. Conclusions

We reported a case of a family with NS. The proband had only a few dental features, whereas her younger sibling presented a series of rare dental manifestations. Prevention strategy and regular dental check-up are essential in patients with NS; therefore, it is important for practitioners involved in oral and maxillofacial care to be aware of the specific features of NS; however, pediatricians and geneticists should pay attention to the oral and maxillofacial management of NS patients.

## Figures and Tables

**Table 1:** Orodental manifestations of patients with Noonan syndrome.

	Patients in This Study		Frequency in Patients with Noonan Syndrome				
Features	P1	P2	Gürsoy et al., 2020 [19] (*n* = 17)	Mallineni et al., 2014 [20] (*n* = 4)	Lutz et al., 2020 [13] (*n* = 10)	Vavetsi et al., 2023 [18] (*n* = 11)	Janas-Naze et al., 2022 [21] (*n* = 42)
delayed dental eruption	X	X	N/A	-	10%	-	N/A
high-arched palate	X	X	76.4%	100%	-	90.9%	N/A
open-bite	-	X	11.7%	-	10%	36.4%	N/A
micrognathia	-	X	-	75%	10%	N/A	N/A
hypoplastic jaw	-	X	N/A	50%	N/A	N/A	N/A
malocclusion	-	X	11.7%	100%	30%	63.6%	N/A
crowded teeth	-	X	-	100%	-	18.2%	N/A
hypodontia	-	-	11.7%	75%	10%	-	N/A
supernumerary teeth	-	-	-	-	-	-	26.2
impacted teeth	-	-	-	100%	N/A	-	23.8
taurodontism	-	-	N/A	100%	-	N/A	N/A
caries	X	X	35.2%	-	-	100%	N/A
enamel hypoplasia	-	-	-	75%	-	N/A	N/A
gingivitis	-	X	35.2%	75%	-	100%	N/A

X, means the manifestation is present; -, means the manifestation is not present; N/A, means data is not available or not determined.

## Data Availability

The data presented in this study are available only on request from the corresponding author due to privacy or ethical reasons.
